# Case report: Benign yet aggressive: Intravascular extension of leiomyoma

**DOI:** 10.1016/j.ijscr.2025.111745

**Published:** 2025-07-27

**Authors:** Ben Ltaief Sarra, Montassar Ghalleb, Mohamed Amine Bouida, Mohamed Maher Rachdi, Mohamed Ali Ayedi, Tarek Ben Dhieb

**Affiliations:** a Surgical Oncology Department, Salah Azaiez Institute of oncology, Tunis, Tunisia; b Faculty of Medicine of Tunis, University Tunis El Manar, Tunis, Tunisia; c Faculty of Medicine of Sfax, University of Sfax, Sfax, Tunisia

**Keywords:** Intravascular leiomyomatosis, Pathology, Misdiagnosis, MRI, CT

## Abstract

**Introduction:**

Intravascular leiomyomatosis (IVL) is a rare, histologically benign condition characterized by the growth of smooth muscle tumors within vascular channels, often mimicking malignant behavior due to intravascular and potential cardiac extension. Despite its benign nature, IVL poses diagnostic and therapeutic challenges due to nonspecific symptoms and understudied malignant potential.

**Case presentation:**

A 47-year-old woman presented with a significant uterine mass and suspected sarcoma, accompanied by tumoral thrombosis extending from the left ovarian to the renal vein. She underwent total hysterectomy with bilateral salpingo-oophorectomy and ligation of the left uterine vein, enabling complete thrombus extraction. Histopathology confirmed IVL, highlighting its unique vascular invasion despite benign histology.

**Clinical discussion:**

IVL's aggressive growth necessitates complete surgical resection to prevent recurrence, as incomplete resection correlates with higher progression risk. Multidisciplinary collaboration is critical, particularly for cardiac involvement, to ensure safe tumor extraction and avoid catastrophic complications. Prognostic studies identify age ≤ 45 and incomplete resection as key recurrence factors, while postoperative hormone therapy (e.g., bilateral oophorectomy) may reduce relapse in estrogen-sensitive cases. Imaging modalities like MRI and CT are pivotal for preoperative planning and differential diagnosis from thrombi or malignancies.

**Conclusion:**

This case underscores IVL's diagnostic complexity and the imperative for radical resection and multidisciplinary management. Increased awareness among gynecologic and cardiovascular specialists is essential to optimize outcomes for this rare, clinically aggressive entity.

## Highlights

• Case Presentation: A 47-year-old woman presented with a uterine mass and tumoral thrombosis extending to the renal vein, managed via total hysterectomy and thrombus extraction. Histopathology confirmed intravascular leiomyomatosis (IVL), a rare benign tumor with vascular invasion.
• Clinical Significance: IVL mimics malignancy due to aggressive vascular growth, posing diagnostic challenges despite its benign histology.
• Management: Radical surgical resection is critical to prevent recurrence, emphasizing multidisciplinary collaboration for safe tumor extraction.
• Awareness: The case underscores the need for heightened vigilance among gynecologic oncologists to recognize IVL and optimize outcomes for this rare, clinically aggressive entity.

## 1 Introduction

Leiomyomas, or uterine fibroids, are benign smooth muscle tumors commonly found in women of reproductive age. Typically, these tumors are confined to the uterine wall and present as non-cancerous growths that are often asymptomatic or associated with symptoms like menorrhagia, pelvic pain, and pressure symptoms [1]. However, in rare cases, leiomyomas can exhibit invasive behavior beyond the uterine confines, a phenomenon known as intravascular leiomyomatosis (IVL). This condition involves the extension of leiomyomatous tissue through the venous system, commonly affecting the pelvic veins and, less frequently, extending into larger venous structures such as the inferior vena cava and renal veins [2].

IVL is a rare condition, affecting approximately 0.25 % to 0.41 % of women with uterine leiomyomas [3]. Despite its rarity, it can pose significant diagnostic and therapeutic challenges due to its potential to mimic more severe conditions. In this report, we present a rare case of IVL in a 47-year-old woman, highlighting its clinical presentation, diagnostic challenges, and management strategies. While leiomyomas are typically benign, this case underscores the potential for such tumors to behave in a more aggressive manner when they invade vascular structures, making accurate diagnosis and appropriate treatment critical.

## 2 Case presentation

A 47-year-old North African woman with a history of irregular menstruation and pelvic discomfort presented with a pelvic mass. The ultrasound initially revealed a large uterine mass measuring approximately 20 cm in size. The mass was hypoechoic, with irregular borders, raising suspicion for a potential uterine sarcoma. On MRI, the mass exhibited a heterogeneous signal on both T1 and T2 sequences. Additionally, tumoral thrombosis was noted within the left ovarian vein, extending into the renal vein, a finding suggestive of more aggressive pathology.

Therapeutic low-molecular-weight heparin (LMWH) was initiated for treatment, and the patient was referred to cardiology for a transthoracic echocardiogram as part of an expanded evaluation. Fortunately, the echocardiogram results were normal, revealing no evidence of thrombosis in the cardiac chambers or the vena cava.

The decision to proceed with surgical removal of the uterus and the mass, rather than performing a biopsy, was made by the tumor board. This approach served as both a therapeutic and diagnostic procedure, allowing us to avoid any potential spread of the disease within the abdominal cavity. After pausing the low-molecular-weight heparin (LMWH) treatment, the patient underwent surgical evaluation.

**Fig. 1:**
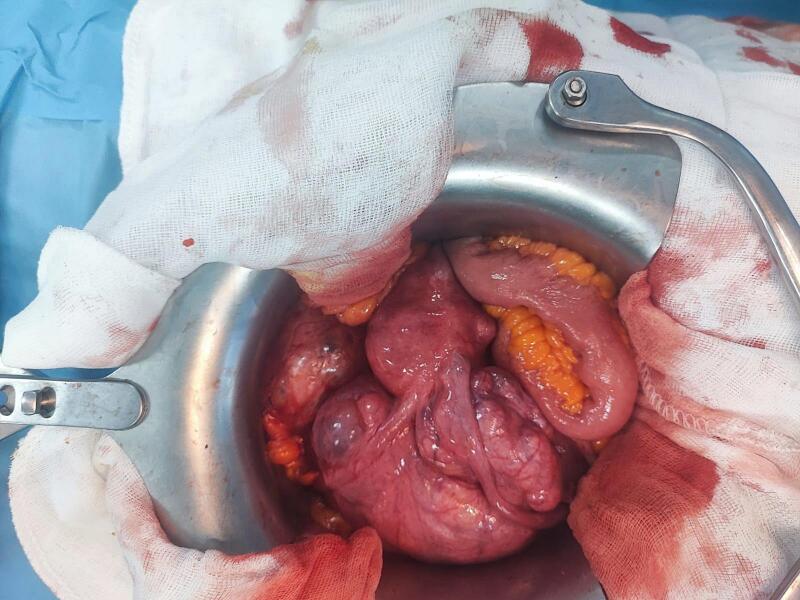
The abdominal cavity before the start of the surgical procedure showing the uterus and the mass.

**Fig. 2:**
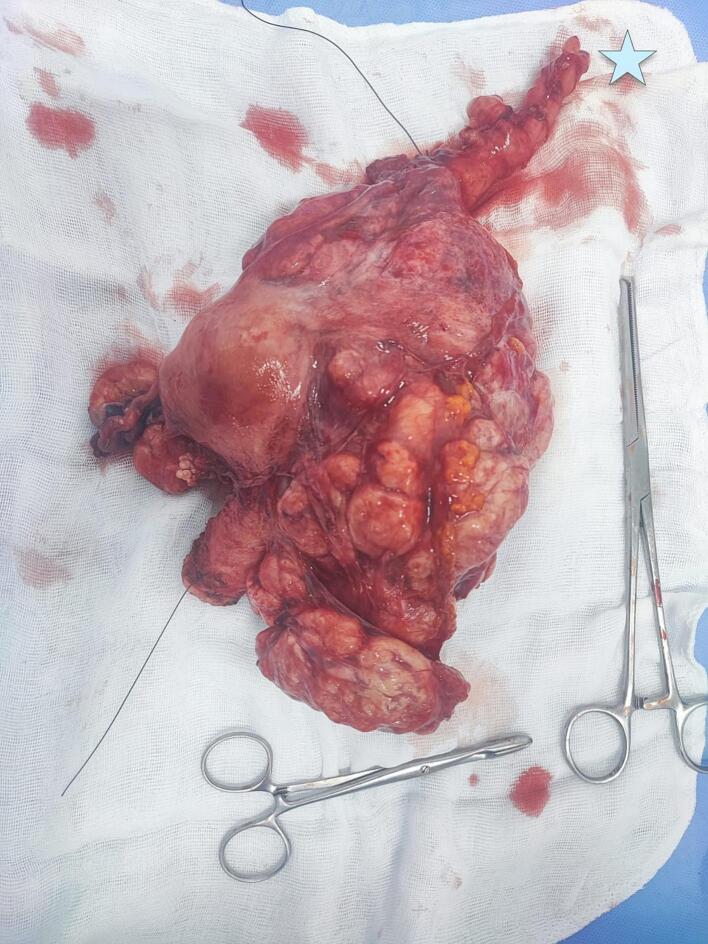
The specimen after total hysterectomy (the blue star showing the engorged left ovarian vein). (For interpretation of the references to colour in this figure legend, the reader is referred to the web version of this article.)

The procedure began with the detachment of the right colon to access the retroperitoneal area, aiming to minimize blood loss by ligating the left ovarian vein as the initial step. Notably, the left uterine vein was found to be significantly engorged, with the thrombus adherent to the vessel wall at the level of the renal vein. The left uterine vein was carefully ligated at the renal vein level, and the tumor thrombus was extracted in its entirety. A total hysterectomy with bilateral salpingo-oophorectomy was subsequently performed. (Fig. 1, Fig. 2).

Preoperatively, the patient's hemoglobin level was 10 g/dL. She received a blood transfusion of one unit during the procedure and another unit postoperatively.

The patient's postoperative recovery was uneventful, and she was discharged five days after admission. During follow-up, no further complications related to the thrombosis or the tumor were observed.

Histological analysis of the excised tissue revealed the presence of smooth muscle cells extending through the venous wall, confirming the diagnosis of intravascular leiomyomatosis. The tumor was composed of well-differentiated smooth muscle cells, resembling a typical leiomyoma, but with notable extension through the venous endothelium. Immunohistochemical staining for smooth muscle actin (SMA) and desmin was positive, further supporting the diagnosis of leiomyoma. There was no evidence of malignancy or sarcomatous transformation.

Timeline of different events is detailed in Table 1.

**Table 1:** Timeline.

Event	Date	Details
Initial symptoms	[Oct/2024]	Patient presented with irregular menstruation and pelvic discomfort.
Ultrasound and MRI	[12/Nov/2024]	20 cm mass suspected to be a uterine sarcoma with tumoral thrombosis extending into renal vein.
Start of LMWH treatment	[13/Nov/2024]	Initiation of low-molecular-weight heparin therapy for thrombosis.
Transthoracic echocardiogram	[21/Nov/2024]	No evidence of cardiac thrombosis.
Surgical decision	[27/Nov/2024]	Tumor board decided to proceed with surgical resection rather than biopsy.
Surgery	[4/Dec/2024]	Total hysterectomy with bilateral salpingo-oophorectomy.
Postoperative recovery & discharge	[9/Dec/2024]	Uncomplicated recovery; discharged 5 days post-surgery.
Histological analysis	[18/Dec/2024]	IVL diagnosed with smooth muscle extension through the venous wall.

## 3 Discussion

Intravascular leiomyomatosis (IVL) is a rare and atypical manifestation of uterine leiomyomas. This condition occurs when smooth muscle tumors extend beyond the uterine wall and invade the venous system, often presenting as a venous thrombus. The etiology of IVL is not fully understood, but two theories exist: the first suggests that the smooth muscle cells of the leiomyoma invade the venous wall either directly or through hematogenous spread, and the second theory assumes that IVL originates from venous vascular smooth muscle cells [4,5].

The clinical presentation of IVL can be highly variable, depending on the location and extent of vascular involvement. In this case, the tumor's extension into the ovarian and renal veins was a key diagnostic feature. Patients may present with symptoms related to venous thrombosis, including leg swelling, pelvic pain, or in more advanced cases, signs of deep vein thrombosis or inferior vena cava syndrome [6]. The association with a uterine mass, particularly when the mass exhibits irregular features, can raise concerns for malignancy, as it did in our patient.

IVL is a rare condition, with fewer than 400 cases reported in the literature since its first description in 1896 [3,7]. Detection of this condition has increased in recent years due to the awareness of this disease.

A critical aspect of diagnosing IVL is distinguishing it from leiomyosarcoma, a more aggressive malignancy. While both conditions may present with uterine masses, IVL is typically associated with benign smooth muscle tumors extending into the venous system, while leiomyosarcoma is a malignant tumor of smooth muscle origin, characterized by a more aggressive behavior and the potential for distant metastasis. Leiomyosarcomas may exhibit significant cellular atypia, necrosis, and a higher mitotic index than benign leiomyomas, including those with intravascular extension. In contrast, IVL typically presents with well-differentiated smooth muscle cells without evidence of malignancy or sarcomatous transformation [8].

Imaging studies, such as MRI and CT scans, are valuable in identifying the mass and evaluating its relationship with adjacent structures, including the vascular system. However, definitive diagnosis often requires histopathological examination of the tumor tissue [9,10]. Immunohistochemistry, specifically for markers such as smooth muscle actin and desmin, p53 or Ki-67 are crucial in confirming the diagnosis of IVL and distinguishing it from other types of venous thrombus or malignancy [8].

The decision to perform a biopsy or surgical intervention depends on several factors, including the size and extent of the tumor, as well as the potential risk of disease spread. In cases where malignancy is suspected, as in our case, surgery may be preferred to avoid any potential spread of the tumor within the abdominal cavity [11].

Surgical intervention is generally the preferred treatment for IVL, typically involving total hysterectomy with bilateral salpingo-oophorectomy. The removal of the ovaries aims to reduce endogenous estrogen levels, which can contribute to tumor growth and recurrence [12]. The choice between laparoscopic and open surgery does not significantly affect recurrence rates and depends on tumor size and the patient's health status [13].

For more advanced stages, early intervention by a multidisciplinary team is recommended [14]. Based on the tumor's vascular extension, a single or double procedure may be necessary. If the tumor extends to the cardiac level, sternotomy followed by abdominal resection may be necessary [15].

While complete resection of IVL generally leads to an excellent prognosis, some patients may experience recurrence, particularly in cases of incomplete excision or extensive vascular involvement. The recommended duration of follow-up after surgery typically involves imaging studies, such as MRI or CT scans, performed every 6 to 12 months for the first 2 years, and annually thereafter for at least 5 years**.** Early recurrence is often detected through radiological monitoring, and patients should be informed of the potential for late recurrence, although it is rare [16].

## 4 Methods

This work has been reported in line with the SCARE, attached as supplementary material [17].

## 5 Conclusion

Intravascular leiomyomatosis is a rare but important consideration in the differential diagnosis of uterine masses, particularly when venous thrombosis is observed. This case highlights the unusual ability of a benign tumor, such as leiomyoma, to invade vascular structures, mimicking more aggressive pathologies. Early recognition and accurate diagnosis are crucial for appropriate management, and surgical resection remains the mainstay of treatment. Awareness of this condition among gynecologic oncologists and radiologists is important to avoid misdiagnosis and to guide optimal treatment strategies.

Given the benign nature of most leiomyomas, it is essential for clinicians to remain vigilant when these tumors exhibit unusual behavior, such as intravascular extension. Further studies are needed to understand the pathophysiology of IVL and to develop guidelines for its management.

## Ethical approval

This case report was reviewed by the ethics committee of Salah Azaiez Institute, which waived the requirement for formal approval because it does not contain identifiable patient information and maintains strict confidentiality.

## Guarantor

Sarra Ben Ltaief.

## Statement of ethics

*Ethics approval and consent to participate.

The approval to publish this case report is not required from the medical and ethics committee, however; this work is done with all due respect to the code of ethics under the supervision of the same committee.

*This case report was reviewed by the ethics committee, which waived the requirement for formal approval because it does not contain identifiable patient information and maintains strict confidentiality. Additionally, the patient provided informed consent for publication of their medical details, aligning with ethical guidelines that permit case report dissemination when patient privacy and rights are protected through explicit consent.

*Written informed Consent for Publication

Written informed consent was obtained from the patient for publication and accompanying images.

A copy of the written consent is available for review by the Editor-in-chief of this journal on request.

## Funding sources

No source of funding.

An IA tool was used to help write this manuscript considering English is not our native language.

## Authors' contribution

Montassar Ghalleb, sarra ben ltaief: Data collection, drafted the manuscript.

Mohamed Amine Bouida, Sarra Ben Ltaief: Data collection review of the literature.

Tarek Ben Dhieb, Mohamed Maher Rachdi: a review of the literature and draft of the manuscript.

Mohamed Ali Ayedi, Montassar Ghalleb: Drafted the manuscript.

## Declaration of competing interest

The authors have no conflicts of interest to declare.

## Data Availability

All data generated or analyzed during this study are included in this article. Further enquiries can be directed to the corresponding author.
